# 

**Published:** 1810-03

**Authors:** 


### NEW MEDICAL PUBLICATIONS.

Observations on the Rupture of the Uterus, on the Snuffles in Infants, and on Mania Lactea. By Thomas Denman, M. D. 8vo. 2s 6d.

Cursory Remarks on Corpulence. By a Member of the Royal Corporation of Surgeons, 2s.

